# Design of a Transdermal Sustained Release Formulation Based on Water-Soluble Ointment Incorporating Tulobuterol Nanoparticles

**DOI:** 10.3390/pharmaceutics14112431

**Published:** 2022-11-10

**Authors:** Noriaki Nagai, Fumihiko Ogata, Saori Deguchi, Aoi Fushiki, Saki Daimyo, Hiroko Otake, Naohito Kawasaki

**Affiliations:** Faculty of Pharmacy, Kindai University, 3-4-1 Kowakae, Higashiosaka 577-8502, Japan

**Keywords:** tulobuterol, nanoparticle, ointment, transdermal delivery system, endocytosis

## Abstract

We aimed to investigate which base was suitable for preparing transdermal formulations incorporating tulobuterol (TUL) nanoparticles (30–180 nm) in this study. Three bases (water-soluble, absorptive, and aqueous ionic cream) were selected to prepare the transdermal formulations, and TUL nanoparticles were prepared with a bead-milling treatment. In the drug release study, the TUL release from the water-soluble ointment was higher than that from the other two ointments. Moreover, the addition of *l*-menthol enhanced TUL nanoparticle release from the ointment, and the rat skin penetration of the TUL water-soluble ointment was also significantly higher than that of the other two ointments. In addition, the drug penetration of the TUL water-soluble ointment with *l*-menthol sustained zero-order release over 24 h, and the skin permeability of TUL increased with TUL content in the ointment. On the other hand, this penetration was significantly inhibited by treatment with a caveolae-mediated endocytosis inhibitor (nystatin). In conclusion, we found that the water-soluble base incorporating TUL nanoparticles and *l*-menthol was the best among those assessed in this study. Furthermore, the pathway using caveolae-mediated endocytosis was related to the skin penetration of TUL nanoparticles in the TUL water-soluble ointment with *l*-menthol. These findings are useful for the design of a transdermal sustained-release formulation based on TUL nanoparticles.

## 1. Introduction

Tulobuterol (TUL), or 2-tert-butylamino-1-(2-chloro-phenyl)-ethanol, is a β2-adrenergic agonist, and transdermal formulations incorporating TUL are used in the treatment of chronic obstructive pulmonary disease (COPD), emphysema, bronchitis, and asthma [1]. Transdermal formulations of TUL can easily maintain plasma drug concentrations at required levels and avoid difficulties associated with oral administration and drug degradation in the gastrointestinal tract; moreover, they provide more consistent serum drug levels, have reduced side effects, and eliminate hepatic first-pass metabolism [2,3,4]. In addition, the administration of transdermal formulations is simple to discontinue, and medication is easily confirmed [5,6]. Although TUL is dissolved in acrylate and rubber in generic transdermal formulations, the original form of commercially available TUL (CA-TUL tape, Hokunalin^®^ Tape, Hisamitsu Pharmaceutical Co., Inc., Tokyo, Japan) is a transdermal patch preparation with a crystal reservoir system, which has been patented. CA-TUL tape contains molecular and crystallized forms of TUL, provides a favorable pharmacokinetic profile, has β2-agonist activity that can be sustained for 24 h, and is widely used by children [1,7,8]. Therefore, CA-TUL tape can be expected to improve adverse drug reactions, making it a useful, long-acting β2-agonist with good adherence that can be applied to children and the elderly [9,10].

On the other hand, recent studies have showed that drug nanoparticles can penetrate deep into the skin, depending on their size [11], and that the transcellular route, intercellular lipid space route, and transappendageal route (hair follicles and sweat glands) are related to drug nanoparticle penetration into the skin [12]. Thus, nanotechnology is a modern and rapidly evolving trend that is being applied in transdermal therapeutic systems (TTS), which include nanocrystals, polymeric nanoparticles, nanovesicles, dendrimers, liposomes, nanomicelles, nanoemulsions, and lipid nanocarriers. Nanovesicles and polymeric nanoparticles possess an advantage over other methods in that they promote transdermal permeation without affecting the skin’s structure [13,14,15]. Moreover, the small size (40 nm–800 nm) of lipid nanocarriers allows them to adhere to the lipid film of the stratum corneum and to increase the number of drug molecules that penetrate into deeper layers of the skin [14,15]. We previously prepared a gel formulation based on *l*-menthol (a skin penetration enhancer) and solid NPs of a drug, demonstrating that gels incorporating drug nanoparticles (NPs) could enhance skin penetration in rat and pig skins [16,17]. Moreover, the reduction in particle size and the utilization of chemical penetration enhancers causes a dramatic increase in the cellular uptake of NPs [16,17,18]. Thus, it is expected that transdermal formulations combined with solid NPs and skin penetration enhancers may be useful as a TTS; however, information on the relationship between bases and NPs in transdermal formulations is not sufficient, and more studies are required.

In this study, we select TUL as a model drug since TUL transdermal formulations are widely used in clinics, and we aim to develop a good transdermal formulation incorporating TUL nanoparticles (TUL-NPs). Ointments are either nongreasy or greasy preparations, depending on the type of base used. Oil-in-water creams are water-soluble and are more cosmetically and aesthetically acceptable than water-in-oil creams. Creams are semisolid emulsions and are usually applied topically as medicated or unmedicated products. We prepare transdermal formulations incorporating TUL-NPs using water-soluble (WS), absorptive (AB), and aqueous ionic cream (AC) ointment bases with and without *l*-menthol (WS, AB, and AC bases). In addition, we investigate the drug release and skin penetration properties of these transdermal formulations.

## 2. Materials and Methods

### 2.1. Animals

Seven-week-old male Wistar rats were purchased from Kiwa Laboratory Animals Co., Ltd. (Wakayama, Japan). They were fed a CE-2 formulation diet (Clea Japan Inc., Tokyo, Japan), and water was provided freely. The rats were housed under normal conditions (light during 7:00 am–7:00 pm; 25 °C). The experiments using rats were approved by the animal care and use committee of Kindai University and were carried out in accordance with the Pharmacy Committee Guidelines.

### 2.2. Chemicals

Pentobarbital was purchased from Sumitomo Dainippon Pharma Co., Ltd. (Toyo, Japan). TUL, cytochalasin D, methyl p-hydroxybenzoate, polyethylene glycol (PEG) 4000, cetyl alcohol, l-menthol, mineral oil, white wax, sodium tetraborate, propylene glycol, beeswax, and sodium dodecyl sulfate were provided by Wako Pure Chemical Industries, Ltd. (Osaka, Japan). Commercially available 0.5 mg TUL tape (CA-TUL, Hokunalin^®^ Tapes 0.5 mg) was obtained from Mylan EPD G.K (Tokyo, Japan). Rottlerin and dynasore were purchased from Nacalai Tesque (Kyoto, Japan), and methylcellulose (MC, SM-4) was obtained from Shin-Etsu Chemical Co., Ltd. (Tokyo, Japan). 2-hydroxypropyl-β-cyclodextrin (HPβCD) was supplied by Nihon Shokuhin Kako Co., Ltd. (Tokyo, Japan), and PEG 400 was provided by Maruishi Pharmaceutical Co., Ltd. (Osaka, Japan). Nystatin and 450 nm pore size MF^TM^ membrane filters were purchased from Sigma-Aldrich Japan (Tokyo, Japan) and Merck Millipore (Tokyo, Japan), respectively. All other chemicals used were of the highest purity commercially available.

### 2.3. Preparation of Ointments Incorporating TUL-NPs

The TUL-NPs were prepared following methods reported in previous studies [18,19,20,21]. Briefly, TUL and methylcellulose (MC) were mixed and milled using a Bead Smash 12 (3000 rpm, 30 s, 4 °C; Wakenyaku Co., Ltd., Kyoto, Japan). Then, 2-hydroxypropyl-β-cyclodextrin (HPβCD) was added and dispersed using distilled water. Thereafter, the dispersions were milled using a Bead Smash 12 (5500 rpm, 60 s × 30 times, 4 °C), providing dispersions containing TUL-NPs. In this study, three bases (water-soluble (WS), absorptive (AB), and aqueous ionic cream (AC) bases) were selected, and the TUL-NP dispersions, as well as the mixtures of TUL, MC, and HPβCD (TUL-MP dispersions), were gelled using the three bases. Table 1 shows the composition of each ointment incorporating TUL. For the WS base, polyethylene glycol (PEG) 4000 and cetyl alcohol were dissolved at 60 °C, and PEG 400 was then added and mixed. The WS/TUL ointments were prepared by the addition of TUL-MPs or TUL-NPs to the WS base. The AB base was prepared as follows: cetyl alcohol, white wax, and mineral oil were dissolved at 60 °C, and subsequently, sodium tetraborate was added. Thereafter, the AB base and TUL (TUL-MPs or TUL-NPs) were mixed (AB/TUL ointments). The AC base was prepared by dissolving cetyl alcohol, beeswax, propylene glycol, and sodium dodecyl sulfate at 60 °C, and the AC/TUL ointments were prepared by the addition of TUL-MPs or TUL-NPs to the AC base. In this study, *l*-menthol was added into these ointments (WS/TUL, AB/TUL, and AC/TUL), and the resulting formulations were denoted as Men-WS/TUL, Men-AB/TUL, and Men-AC/TUL.

### 2.4. Measurement of TUL Particles

The size of the TUL-MPs was measured using a SALD-7100 laser diffraction particle size analyzer (Shimadzu Corp., Kyoto, Japan), and the size of the TUL-NPs was determined using both a SALD-7100 and a NANOSIGHT LM10 dynamic light-scattering analyzer (QuantumDesign Japan, Tokyo, Japan). The measurement conditions for the SALD-7100 analyzer were a maximum value of scattered light intensity in the range of 40–60% and a refractive index of 1.60 ± 0.10i. The measurement conditions for the NANOSIGHT LM10 analyzer were a wavelength of 405 nm (blue), a time of 60 s, and a viscosity of 1.27 mPa∙s. In addition, the number of TUL-NPs was also detected using a NANOSIGHT LM10 analyzer. Images of ointments incorporating TUL were captured using a scanning probe microscope (SPM). The SPM images were captured with a SPM-9700 (Shimadzu Corp., Kyoto, Japan) instrument. During SPM measurements, the ointments were washed with distilled water, and the isolated TUL particles were measured.

### 2.5. Drug Solubility of TUL Ointments

Dissolved TUL and TUL-NPs in the ointments were isolated using centrifugation at 100,000× *g* (Beckman Optima^TM^ MAX-XP Ultracentrifuge, Beckman Coulter, Osaka, Japan). Isolated TUL was dissolved using methanol, and the content was measured in order to calculate the solubility of TUL in the ointments. The TUL contents were measured using the HPLC method described below.

### 2.6. Viscosity of the Ointments

A Brookfield digital viscometer was used to measure the viscosity of the TUL ointments, as presented in Table 1 (Brookfield Engineering Laboratories, Inc., Middleboro, MA, USA).

### 2.7. Stability of the TUL Ointments

As presented in Table 1, an amount of 0.3 g of each TUL ointment was placed in a beaker, and the ointments were kept in a refrigerator (4 °C) with a lid to keep them from drying out. After 1 month, ointments were removed from beakers, and the changes in particle size and number, as well as content, of TUL were measured with a NANOSIGHT LM10 instrument and the HPLC method described below. When measuring particle characteristics of the ointments, the ointments were distributed in 100 mL of water since accurate particle size could not be measured in the ointment state.

### 2.8. Drug Release from TUL Ointments

Franz diffusion cells were used to measure the release of TUL from the ointments as previously reported [16,17,18]. Briefly, 12.2 mL of phosphate-buffered solution (pH 7.2) was transferred into a reservoir chamber and thermoregulated at 37 °C. MF^TM^ membrane filters (450 nm pore size) were set in a Franz diffusion cell. Then, O-ring flanges (1.6 cm i.d.) were placed on the filters, and 0.3 g of each prepared ointment or CA-TUL tape was spread uniformly over the filters. In the experiment, 100 μL of sample solution was withdrawn from the reservoir chamber at 0.5, 1, 3, 6, and 24 h and supplemented with the same volume of pH 7.2 phosphate-buffered solution. The collected samples were used to measure particle size and number, as well as content, of TUL with a NANOSIGHT LM10 analyzer and the HPLC method described below. The area under the TUL concentration–time curve (AUC_Release_) was analyzed according to the trapezoidal rule to the last TUL measurement point (24 h).

### 2.9. In Vitro Transdermal Penetration of Ointments Incorporating TUL

The in vitro transdermal penetration of the ointments was evaluated following the methods described in previous studies [16,17,18]. The hair on the abdominal areas of 7-week-old Wistar rats was removed on the day prior to the experiment, and the rats were euthanized by injection with a lethal dose of pentobarbital on the day of the experiment. Thereafter, the abdominal area was collected and set in a Franz diffusion cell. A total of 12.2 mL of phosphate-buffered solution (pH 7.2) was transferred into a reservoir chamber and thermoregulated at 37 °C. Then, O-ring flanges (1.6 cm i.d.) were placed on filters, and 0.3 g of each prepared ointment or CA-TUL tape was spread uniformly over the filters. In the experiment, 100 μL of sample solution was withdrawn from the reservoir chamber at 0.5, 1, 3, 6, and 24 h and supplemented with the same volume of pH 7.2 phosphate-buffered solution. The collected samples were used to measure the particle size and number, as well as content, of TUL with a NANOSIGHT LM10 instrument and the HPLC method described below. The trapezoidal rule to the last TUL measurement point (24 h) was used to analyze the area under the penetrated TUL concentration–time curves (AUC_Skin_), and pharmacokinetic parameters were analyzed according to Equations (1) and (2) using a nonlinear least-squares computer program (MULTI) [22].

(1) $$\;D=\frac{\delta^{2}}{6\tau }\;$$

(2) $$J_{c}=\frac{Q}{A\cdot \left(t-\tau \right)}=\frac{D\cdot K_{m}\cdot C_{c}}{\delta }{=K}_{p}\cdot C_{c}\;$$

where *J_c_*, *K_m_*, *K_p_*, *D*, *τ*, *A*, *δ*, *Q_t_*, and *C_c_* are the penetration rate, skin coefficient, preparation partition coefficient, diffusion constant within the skin, penetration coefficient through the skin, lag time, effective area of the skin (2 cm^2^), thickness of the skin (0.071 cm, *n* = 5), and amount of TUL in the reservoir solution at time t, respectively.

Nystatin (54 μM) [23], dynasore (40 μM) [24], rottlerin (2 μM) [16,25], and cytochalasin D (10 μM) [23] were used to inhibit caveolae-mediated endocytosis (CavME), clathrin-mediated endocytosis (CME), micropinocytosis (MP), and phagocytosis, respectively. These pharmacological inhibitors were dissolved in 0.5% DMSO and applied to the removed skin 1 h prior to the application of the transdermal formulations.

### 2.10. Measurement of TUL by HPLC Method

TUL was measured using an HPLC method. A Shimadzu LC-20AT system equipped with an SIL-20AC auto-injector and a CTO-20 A column oven (Shimadzu Corp.) was used to measure the TUL content. TUL was dissolved in methanol containing internal standard (1 μg/mL methyl p-oxybenzoate), and 10 μL was injected into the HPLC. The column used was an Inertsil^®^ ODS-3 column (3 μm, column size: 2.1 mm × 50 mm; GL Science Co., Inc., Tokyo, Japan), and 0.02 M potassium dihydrogen phosphate:acetonitrile (87:13, *v*/*v*) was utilized as the mobile phase at 0.25 mL/min and 35 °C. TUL was detected at 211 nm.

### 2.11. Statistical Analysis

Statistical analyses were performed using Dunnett’s multiple comparisons (one-way analysis of variance), and *p* < 0.05 was chosen as the significance level. The data are expressed as means ± standard error (SE) of the mean.

## 3. Results

### 3.1. Evaluation of Ointments Incorporating TUL-NPs

**Figure 1:**
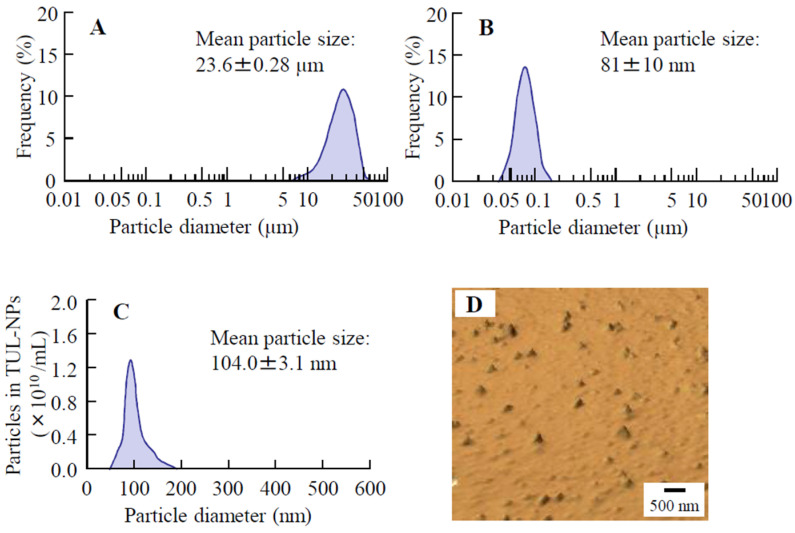
Particle size frequencies and SPM image of TUL in TUL-MP and TUL-NP dispersions. Particle distribution of TUL-MP (**A**) and TUL-NP (**B**) dispersions using laser diffraction particle size analyzer. Particle distribution (**C**) and SPM image (**D**) of TUL-NPs in TUL-NP dispersions using dynamic light-scattering analyzer and SPM-9700 instrument, respectively. Particle sizes of bead-mill-treated TUL particles were on the nanoscale, i.e., 30–180 nm.

**Figure 2:**
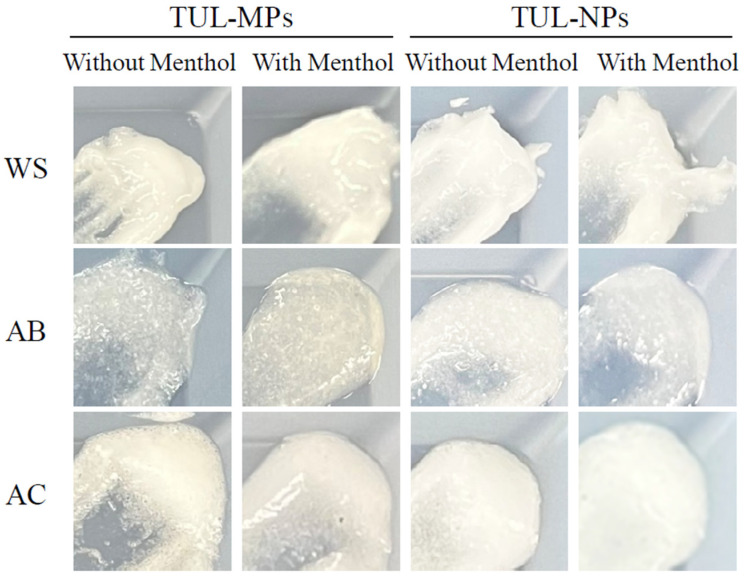
Digital images of ointments incorporating TUL-MPs and TUL-NPs. The compositions of ointments incorporating TUL-MPs and TUL-NPs are presented in Table 1.

Figure 1 shows the particle size distribution of TUL particles with or without bead-milling treatment. The mean TUL particle size without bead-milling treatment was 23.6 ± 0.28 μm (Figure 1A). Alternately, the size distribution of TUL particles with the bead-milling treatment was 30–180 nm (Figure 1B,C), and AFM imaging of TUL using SPM showed that the TUL particles with bead-milling treatment were uniformly dispersed with no large agglomerates. Figure 2 shows images of the ointments incorporating TUL-MPs and TUL-NPs. Although the TUL particles were observed in the ointments incorporating TUL-MPs, the TUL particles could not be visually confirmed in the ointments incorporating TUL-NPs. The ointments incorporating TUL-NPs appeared whitish in comparison with the ointments incorporating TUL-MPs. Table 2 shows the mean particle size, the solubility, and the viscosity of ointments incorporating TUL-MPs and TUL-NPs. The particle sizes in the ointments incorporating TUL-NPs remained on the nanoscale. The amounts of dissolved TUL in the WS/TUL-NP, AB/TUL-NP, and AC/TUL-NP ointments were higher than those in the corresponding ointments incorporating TUL-MPs. On the other hand, significant differences were not observed between the solubility levels in the WS/TUL, AB/TUL, and AC/TUL ointments with and without *l*-menthol. The viscosities were also similar in the ointments incorporating TUL-MPs and TUL-NPs, with the following order of viscosity: WS/TUL > AC/TUL > AB/TUL. Although the viscosities were similar in the AB/TUL and AC/TUL ointments with and without *l*-menthol, the addition of *l*-menthol decreased the viscosity in the WS/TUL ointments. In this study, we measured concentration and particle size 1 month after preparation. The TUL concentrations in the WS/TUL-NP, AB/TUL-NP, and AC/TUL-NP ointments were not changed and remained nanosized. The mean particle sizes in the WS/TUL-NP, Men-WS/TUL-NP, AB/TUL-NP, Men-AB/TUL-NP, AC/TUL-NP, and Men-AC/TUL-NP ointments were 121, 118, 126, 120, 113, and 119 nm, respectively.

### 3.2. Drug Release of TUL in WS/TUL, AB/TUL, and AC/TUL Ointments with and without l-Menthol

**Figure 3:**
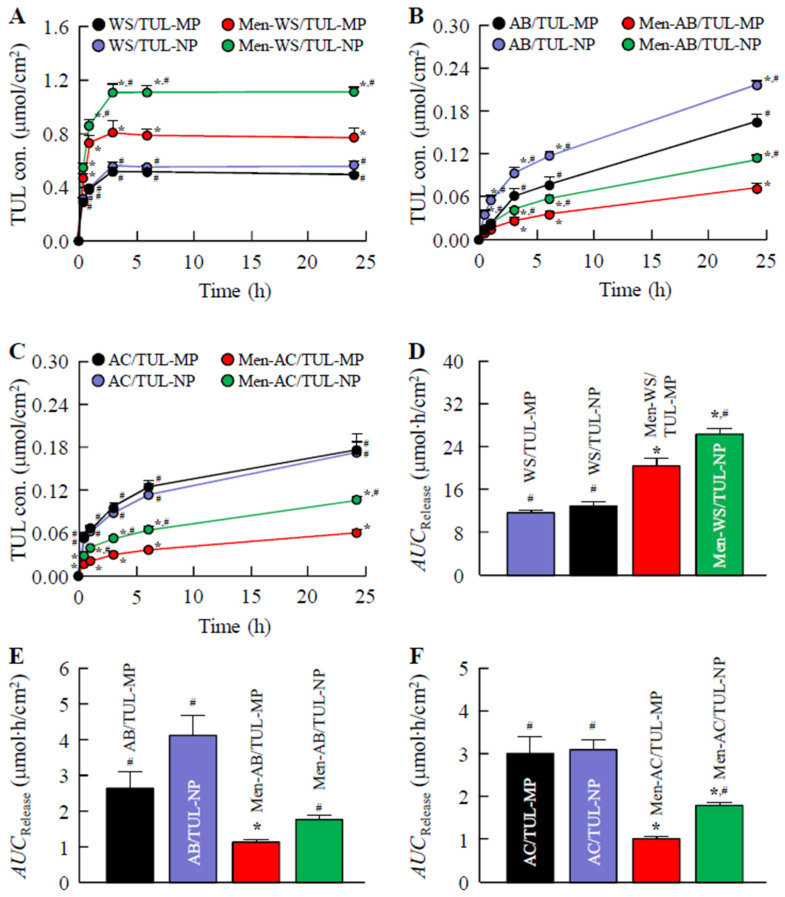
TUL release from ointments incorporating TUL-MPs and TUL-NPs through 450 nm pore membranes. Release of TUL from WS/TUL (**A**); AB/TUL (**B**); and AC/TUL (**C**) ointments with and without *l*-menthol through membranes. *AUC*_Release_ of WS/TUL (**D**); AB/TUL (**E**); and AC/TUL (**F**) ointments with and without *l*-menthol through 450 nm pore membranes. The compositions of ointments incorporating TUL-MPs and TUL-NPs are presented in Table 1. Results are means ± SE; *n* = 6–10. * *p* < 0.05 vs. MP formulation for each category. ^#^ *p* < 0.05 vs. MP formulation with *l*-menthol for each category. The TUL release values from WS/TUL ointments were higher than those from AB/TUL and AC/TUL ointments, and the combination of NPs and *l*-menthol enhanced drug release from the WS base.

Figure 3 shows the release profiles of TUL from the WS/TUL, AB/TUL, and AC/TUL ointments with and without *l*-menthol. The drug release values from the WS/TUL ointments were higher than those of the ointments with AB and AC bases. The *AUC*_Release_ values were similar for the WS/TUL and AC/TUL ointments incorporating both MPs and NPs. On the other hand, the TUL release from the AB/TUL-NP ointment was higher than that of the AB/TUL-MP ointment. The addition of *l*-menthol decreased the TUL release from the AB and AC bases. In contrast to the results for the AB and AC bases, the TUL release from the WA base was enhanced by the addition of *l*-menthol. In the AB/TUL-NP and AC/TUL-NP ointments with and without *l*-menthol, the numbers of TUL-NPs were low (AB/TUL-NP, 5.14 ± 0.38 × 10^5^; Men-AB/TUL-NP, 1.10 ± 0.21 × 10^6^; AC/TUL-Ns, 2.94 ± 0.58 × 10^5^; Men-AC/TUL-NP, 2.79 ± 0.85 × 10^6^ (particles/mL)). However, more TUL-NPs were released into the reservoir chamber by the WS/TUL-NP and Men-WS/TUL-NP ointments. The particle size frequencies (particle numbers) of the released TUL-NPs were 220.8 ± 11.5 nm (2.22 ± 0.15 × 10^8^ particles/mL) and 200.2 ± 10.2 nm (5.39 ± 0.93 × 10^8^ particles/mL), as detected in the reservoir chamber 24 h after the application of the WS/TUL-NP and Men-WS/TUL-NP ointments, respectively.

### 3.3. Transdermal Delivery of TUL in WS/TUL, AB/TUL, and AC/TUL Ointments with and without l-Menthol

**Figure 4:**
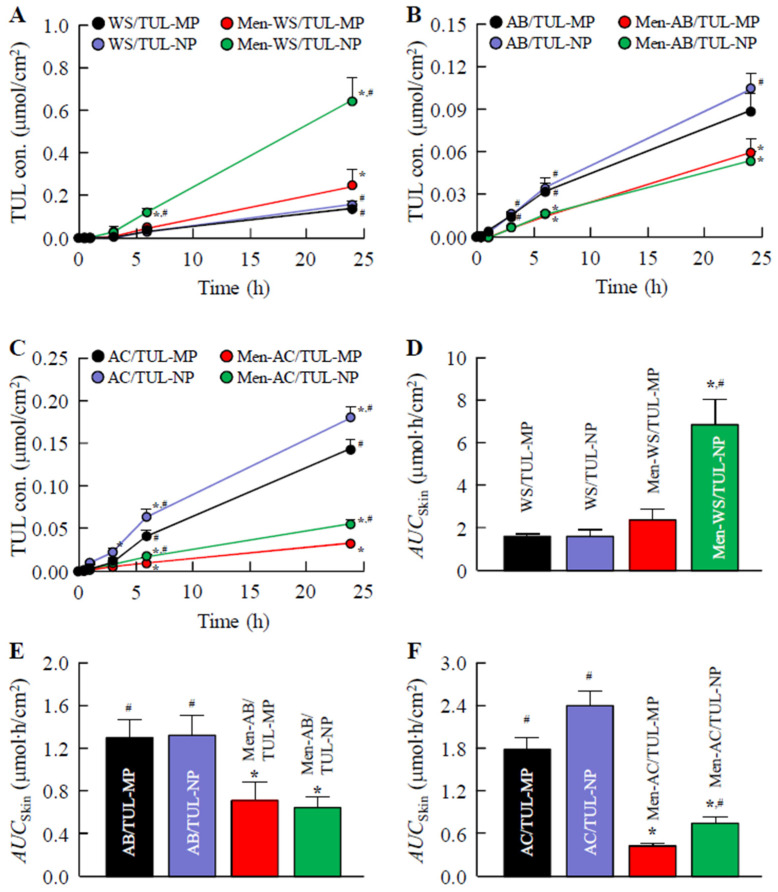
Transdermal penetration of TUL in ointments incorporating TUL-MPs and TUL-NPs. Penetration of WS/TUL (**A**); AB/TUL (**B**); and AC/TUL (**C**) ointments with and without *l*-menthol through rat skin. *AUC*_Release_ values of WS/TUL (**D**); AB/TUL (**E**); and AC/TUL (**F**) ointments with and without *l*-menthol through rat skin. The compositions of ointments incorporating TUL-MPs and TUL-NPs are presented in Table 1. Results are means ± SE; *n* = 6–8. * *p* < 0.05 vs. MP formulation for each category. ^#^ *p* < 0.05 vs. MP formulation with *l*-menthol for each category. TUL penetration amounts of the WS/TUL ointments were higher in comparison with AB/TUL and AC/TUL ointments, and the combination of NPs and *l*-menthol in the WS base significantly increased skin penetration.

**Figure 5:**
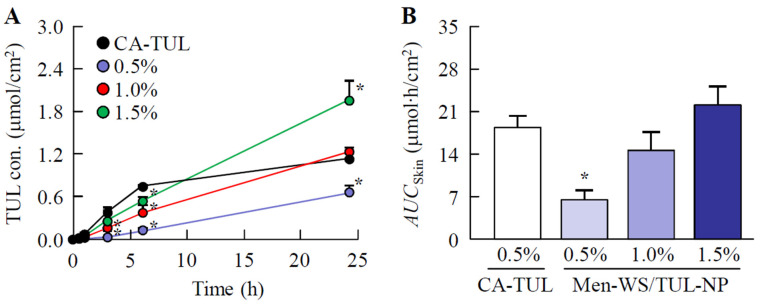
Changes in the transdermal penetration of TUL in Men-WS/TUL ointments containing 0.5–1.5% TUL-NPs. Changes in TUL profiles (**A**) and *AUC*_Skin_ values (**B**) of Men-WS/TUL-NP ointments (0.5–1.5% TUL). Results are means ± SE; *n* = 6–8. * *p* < 0.05 vs. CA-TUL tape for each category. The transdermal penetration of TUL in Men-WS/TUL-NP ointment was enhanced with TUL content, and the penetration profile linearly increased.

Figure 4 shows the skin penetration profiles of the WS/TUL, AB/TUL, and AC/TUL ointments with and without *l*-menthol, and Table 3 shows the pharmacokinetic parameters estimated from the data in Figure 4. The *AUC*_Skin_ values for the WS base were higher than those of the AB and AC bases. In the AB/TUL and AC/TUL ointments, the skin penetration of TUL in the ointment was decreased by the addition of *l*-menthol, although *l*-menthol enhanced the skin penetration of TUL in the WS/TUL ointment. Although the *AUC*_Skin_ values in the AB/TUL ointments incorporating TUL-NPs and TUL-MPs were not significantly different, the *AUC*_Skin_ values for the Men-WS/TUL-NP and Men-AC/TUL-NP ointments were significantly higher than those of the corresponding ointments containing TUL-MPs. In particular, the skin penetration of the Men-WS/TUL-NP ointment was greater than that of the Men-WS/TUL-MP ointment, and the *AUC*_Skin_ value of the Men-WS/TUL-NP ointment was 2.87-fold that of the Men-WS/TUL-MP ointment. In addition, the *J*_c_, *K*_m_, and *K*_p_ values of the Men-WS/TUL-NP ointment were also significantly enhanced in comparison with the Men-WS/TUL-MP ointment. Figure 5 shows the relationship between TUL content and skin penetration in the Men-WS/TUL-NP ointment. The skin penetration of TUL in the Men-WS/TUL-NP ointment linearly increased with TUL content for 24 h, and sustained penetration was observed in comparison with CA-TUL tape since the penetration rate of TUL in CA-TUL tape decreased after 6 h. The skin penetration of CA-TUL tape was higher than that of the Men-WS/TUL-NP ointment at the same content, although the *AUC*_Skin_ values in ointments incorporating 1.0–1.5% TUL-NPs were similar to that of CA-TUL tape. In addition, we investigated whether the particles penetrated through the skin tissue by the measurement of particles in the receiving phase of the permeation study. In contrast to the results of the drug release experiments using membrane filters, the number of TUL-NPs was not quantified in the reservoir chamber for the in vitro studies using rat skin treated with Men-WS/TUL-NP ointment.

### 3.4. Effect of Energy-Dependent Endocytosis on Transdermal Pathway of TUL-NPs in Men-WS/TUL-NP Ointment

**Figure 6:**
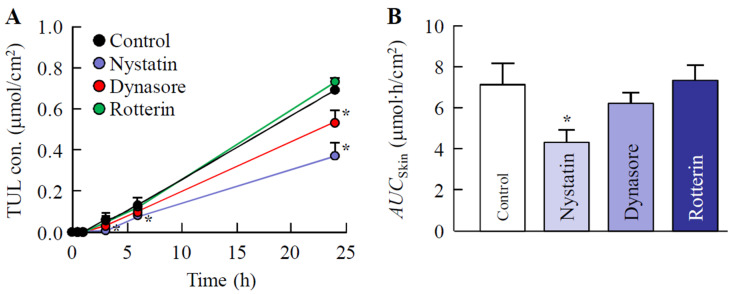
Effect of energy-dependent endocytosis on the transdermal penetration of TUL in the Men-WS/TUL-NP ointment. Changes in the TUL profiles (**A**) and *AUC*_Skin_ values (**B**) of the Men-WS/TUL-MP ointment after treatment with endocytosis inhibitors (nystatin, dynasore, and rottlerin). The composition of the Men-WS/TUL-NP ointment is presented in Table 1. Results are means ± SE; *n* = 6–8. * *p* < 0.05 vs. control for each category. The transdermal penetration of TUL-NPs was significantly inhibited by treatment with CavME inhibitor (nystatin).

Figure 6 shows the skin penetration of TUL-NPs from the Men-WS/TUL-NP ointment into skin treated with endocytosis inhibitors. No significant difference was observed in the transdermal penetration of skin treated with or without rottlerin. On the other hand, transdermal penetration in skin treated with dynasore tended to decrease in comparison with the control group. In addition, nystatin treatment significantly inhibited the transdermal penetration of the Men-WS/TUL-NP ointment. The *AUC*_Skin_ value in skin treated with nystatin was 60.7% that of the control group.

## 4. Discussion

The objective of the present study was to evaluate the suitability of WS, AB, and AC bases in the preparation of transdermal formulations incorporating TUL-NPs. We found that the skin penetration of TUL in the Men-WS/TUL-NP ointment linearly increased with TUL content for 24 h, and the sustained penetration was higher than that of CA-TUL tape. In addition, we showed that the CavME pathway was related to the high level of skin penetration for the Men-WS/TUL-NP ointment.

First, we attempted to prepare TUL-NPs. Previous studies have shown that the selection of additives is important for effective milling and that MC enhances the milling efficiency in bead-milling treatment. In addition, HPβCD prevents the aggregation of drug NPs [16,17,18,19,20,21]. According to these reports, we prepared TUL-NPs using additives (MC and HPβCD) and bead-milling treatment and succeeded in providing dispersions containing TUL-NPs (Figure 1). Following that, we investigated which types of bases were suitable for the development of transdermal formulations incorporating TUL-NPs since the selection of an appropriate formulation base is essential for the ease of application of nanoparticles, as well as the enhancement of dermal and transdermal delivery [26]. Ointments are denoted as oil-based, emulsion-based, and water-soluble-based, and it is suggested that the emulsion-based or water-soluble-based ointments are suitable for maintaining drugs in ointments for solid NPs since hydrophobic drugs are dissolved by oil-based ointments. Ahmed et al. [27] reported that WS, AB, and AC bases provided the best release of a hydrophobic drug (mefenamic acid) among cream and ointment bases. In addition, our previous studies using Carbopol and MC gel bases have shown that drug release decreases with higher polymer concentrations [28,29]. In summary, we selected WS, AB, and AC bases to prepare transdermal formulations incorporating TUL-NPs in this study and determined the lowest concentration of each polymer needed to form gels (Table 1). In any of the bases, the TUL size particle in ointments incorporating TUL-NPs was on the nanoscale, i.e., 30 nm–200 nm (Table 2). In addition, the addition of *l*-menthol did not affect the drug solubility of the TUL ointments (Table 2).

Thereafter, we investigated the drug release and skin penetration of these ointments. The TUL release values from the WS base were higher than those of the AB and AC bases (Figure 3). Moreover, the release of TUL from ointments incorporating TUL-NPs tended to be higher than those incorporating TUL-MPs (Figure 3). *l*-menthol decreased TUL release in the creams (the AB and AC bases); however, the TUL release from hydrophilic ointments (the WA base) was increased by the addition of *l*-menthol. *l*-menthol is a hydrophobic drug; therefore, it was hypothesized that the affinity of creams and *l*-menthol would be higher than that of hydrophilic ointments and that *l*-menthol had a high affinity for TUL. A high affinity between *l*-menthol, TUL, and the bases (AB and AC bases) could cause a decrease in TUL release from the ointment. In addition, it was suggested that the enhanced TUL release from the WC base was due to the low affinity between *l*-menthol and the WC base in comparison with that of the AB and AC bases. Moreover, the viscosity of the WS base was significantly higher than that of the AC and AB bases, and the addition of *l*-menthol decreased the viscosity of the WS base incorporating TUL (Table 2). Our previous studies have shown that enhanced viscosity attenuates the drug release of NPs from ointments [28,29]. From these results, the decreased viscosity could also be related to the enhanced TUL release from the WC base as a result of the addition of *l*-menthol.

It is essential to understand the factors influencing the skin permeability of nanoparticles to provide safe and efficient therapeutic applications. A previous study reported that skin penetration was influenced by the physicochemical characteristics of nanocarriers, such as composition, size, shape, and surface chemistry, as well as skin features [30]. In this study, the skin permeability levels of ointments incorporating TUL were also similar to the drug release tendencies of corresponding ointments, although the skin penetration of the Men-WS/TUL-NP ointment was significantly higher in comparison with the other ointments (Figure 4). It is known that particles over 100 nm in size cannot penetrate skin tissue [18,31], although the addition of *l*-menthol alters the barrier properties of the stratum corneum and causes reversible disruption of the lipid domains since menthol preferentially distributes into the intercellular spaces of the stratum corneum. Thus, the combination of *l*-menthol increases the skin absorption of NPs [18,32,33]. In addition, the release amounts of TUL-NPs from the WS/TUL-NP ointments with and without *l*-menthol were higher than those of the AB and AC bases. In summary, the high drug release from the base and the decrease in barrier function of the stratum corneum resulting from *l*-menthol may cause the high skin permeability found for the Men-WS/TUL-NP ointment. Moreover, the release of NPs from bases incorporating TUL-NPs was observed in the drug release examination; however, TUL-NPs were not detected in the in vitro studies using rat skin treated with the Men-WS/TUL-NP ointment. These results suggest that the released TUL-NPs from the Men-WS/TUL-NP ointment were dissolved in the skin permeation process.

It is important to clarify the levels of absorption and sustained drug release compared with those of CA-TUL tape. We have previously reported that the percutaneous absorption of a transdermal formulation based on drug NPs is higher than that in transdermal formulations based on dissolved and MP drugs [16,17,18]. In contrast to the data from our previous studies, the transdermal penetration of the Men-WS/TUL-NP ointment was less than that of CA-TUL tape at the same content. CA-TUL tape is a patch preparation, and the differences between patches and ointments may relate to the transdermal penetration of TUL. On the other hand, the skin penetration of TUL in the Men-WS/TUL-NP ointment linearly increased with TUL content, and the sustained efficiency was higher than that of CA-TUL tape (Figure 5). In addition, the skin penetration of the Men-WS/TUL-NP ointment containing 1.5% TUL-NPs was significantly higher than that of CA-TUL tape at 24 h in the in vitro transdermal penetration test using rat skin (Figure 5). Considering rheological properties, drug release, and skin penetration, the WS base incorporating TUL-NPs and *l*-menthol (the Men-WS/TUL-NP ointment) was the best among the studied formulations.

Furthermore, we demonstrated the pathway for the transdermal absorption of TUL-NPs in the Men-WS/TUL-NP ointment. The endocytosis of nanomedicine has drawn tremendous interest in the last decade, and endocytosis is one of the functions for breaching tissue barriers, resulting in the efficient delivery of nanoparticles [34,35]. In particular, it has been reported that energy-dependent endocytosis is the major route by which nanomedicines are transported across membranes [36,37,38,39]. Energy-dependent endocytosis processes related to the uptake of NPs are classified as being CavME, CME, or MP, and the sizes of the vesicles vary with the specific pathway of the endocytic processes, i.e., the sizes for the CavME, CME, and MP pathways are <80 nm, <120 nm, and 100 nm–5 μm, respectively [40]. The particle size of the TUL-NPs in the Men-WS/TUL-NP ointment was 30 nm–200 nm (Table 2), and we showed that nystatin attenuated the skin penetration of TUL in the Men-WS/TUL-NP ointment (Figure 6). In summary, we hypothesized that the CavME pathway was the main skin-related pathway of the TUL-NPs in the Men-WS/TUL-NP ointment. In addition, the released TUL-NPs from the Men-WS/TUL-NP ointment could be dissolved in this process, since the TUL-NPs were not detected in the in vitro studies using rat skin treated with the Men-WS/TUL-NP ointment.

Further studies are needed to clarify the CavME pathway and to obtain more pharmacokinetic data on the in vivo percutaneous absorption of TUL-NPs in the Men-WS/TUL-NP ointment in animal models. In addition, it is important to elucidate the usefulness of the Men-WS/TUL-NP ointment. Therefore, we plan to investigate the therapeutic effects of Men-WS/TUL-NP ointments (0.5–1.5% TUL) on the long-term management of asthma and COPD using an animal model.

## 5. Conclusions

**Figure 7:**
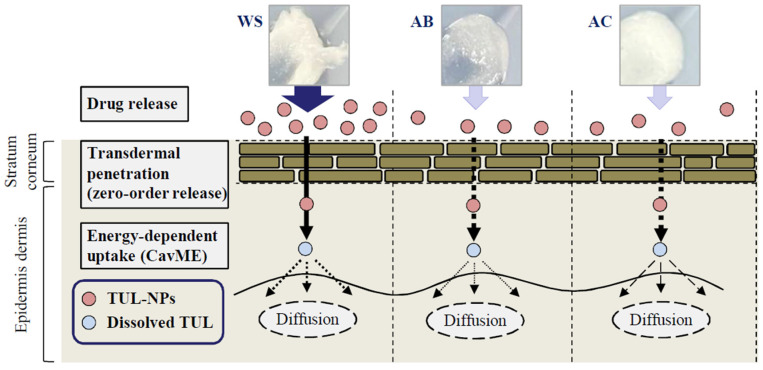
Suggested mechanism for the transdermal penetration process of TUL from ointment bases incorporating TUL-NPs and *l*-menthol.

We produced transdermal formulations based on WS, AB, and AC bases incorporating TUL-NPs and evaluated which bases were suitable for preparing transdermal formulations incorporating TUL-NPs. We found that the WS base incorporating TUL-NPs and *l*-menthol (the Men-WS/TUL-NP ointment) was the best among those assessed in this study. In addition, we showed that the penetration of TUL from the Men-WS/TUL-NP ointment sustained zero-order release over 24 h, and its skin permeability increased with TUL content in the Men-WS/TUL-NP ointment. Furthermore, the CavME pathway was related to the skin penetration of TUL-NPs in the Men-WS/TUL-NP ointment (Figure 7). These findings may be utilized to improve the transdermal delivery of TUL in future studies.

## Figures and Tables

**Table 1:** Compositions of WS/TUL, AB/TUL, and AC/TUL ointments with and without *l*-menthol.

Ointment	Content (%*w*/*w*)					
	WS/TUL	Men-WS/TUL	AB/TUL	Men-AB/TUL	AC/TUL	Men-AC/TUL
TUL	0.2	0.2	0.2	0.2	0.2	0.2
MC	0.05	0.05	0.05	0.05	0.05	0.05
HPβCD	0.5	0.5	0.5	0.5	0.5	0.5
PEG 4000	43.8	43.8	-	-	-	-
PEG 400	43.8	43.8		-	-	-
Cetyl alcohol	0.4	0.4	12.5	12.5	15	15
Mineral oil	-	-	56	56	-	-
White wax	-	-	12	12	-	-
Sodium tetraborate	-	-	0.5	0.5	-	-
Propylene glycol	-	-	-	-	10	10
Beeswax	-	-	-	-	1	1
Sodium dodecyl sulfate	-	-	-	-	2	2
*l*-menthol	-	2	-	2	-	2
Distilled water ad.	100	100	100	100	100	100

**Table 2:** Changes in particle size, solubility, and viscosity of WS/TUL, AB/TUL, and AC/TUL ointments with and without *l*-menthol.

Ointment		Mean Particle Size (μm)	Solubility (μM)	Viscosity (mPa∙s)
WS/TUL	WS/TUL-MP	28.0 ± 0.31	139 ± 7.9	1476 ± 83
	WS/TUL-NP	0.113 ± 0.0038	192 ± 9.3 *^,#^	710 ± 47 *^,#^
	Men-WS/TUL-MP	27.5 ± 0.29	139 ± 8.5	1466 ± 86
	Men-WS/TUL-NP	0.113 ± 0.0033	193 ± 9.0 *^,#^	747 ± 45 *^,#^
AB/TUL	AB/TUL-MP	28.3 ± 0.32	112 ± 6.9	79.6 ± 7.8
	AB/TUL-NP	0.119 ± 0.0038	189 ± 9.2 *^,#^	76.3 ± 7.9
	Men-AB/TUL-MP	27.7 ± 0.29	113 ± 8.1	77.5 ± 7.9
	Men-AB/TUL-NP	0.116 ± 0.0035	185 ± 8.8 *^,#^	70.2 ± 8.4
AC/TUL	AC/TUL-MP	29.2 ± 0.36	116 ± 6.8	599 ± 37
	AC/TUL-NP	0.110 ± 0.0039	186 ± 7.9 *^,#^	597 ± 35
	Men-AC/TUL-MP	28.1 ± 0.28	120 ± 7.0	606 ± 39
	Men-AC/TUL-NP	0.112 ± 0.0036	188 ± 9.1 *^,#^	605 ± 38

The compositions of ointments containing TUL-MPs and TUL-NPs are presented in Table 1. *n* = 6–10. * *p* < 0.05 vs. MP formulation for each category. ^#^ *p* < 0.05 vs. MP formulation with *l*-menthol for each category.

**Table 3:** Pharmacokinetic analysis of TUL transdermal formulations in the in vitro penetration of rat skin.

Ointment		*J*_c_ (×10^−2^ μmol/cm^2^/h)	*K*_p_ (×10^−3^ cm/h)	*K*_m_	*τ* (h)	*D* (×10^−3^ cm^2^/h)
WS/TUL	WS/TUL-MP	0.09 ± 0.01 ^#^	0.13 ± 0.01 ^#^	0.01 ± 0.01 ^#^	0.09 ± 0.03 ^#^	0.25 ± 0.17 ^#^
	WS/TUL-NP	0.10 ± 0.02 ^#^	0.14 ± 0.02 ^#^	0.01 ± 0.01 ^#^	1.17 ± 0.01	0.14 ± 0.01 ^#^
	Men-WS/TUL-MP	0.62 ± 0.10 *	0.85 ± 0.14 *	0.07 ± 0.01	1.01 ± 0.07	0.83 ± 0.05 *
	Men-WS/TUL-NP	2.79 ± 0.49 *^,#^	3.82 ± 0.68 *^,#^	0.36 ± 0.07 *^,#^	1.13 ± 0.02	0.74 ± 0.02 *
AB/TUL	AB/TUL-MP	0.07 ± 0.05	0.10 ± 0.07	0.03 ± 0.02	0.90 ± 0.43	3.70 ± 2.11
	AB/TUL-NP	0.11 ± 0.08	0.16 ± 0.11	0.02 ± 0.01	0.29 ± 0.14	3.65 ± 2.08
	Men-AB/TUL-MP	0.05 ± 0.02	0.07 ± 0.05	0.01 ± 0.01	0.43 ± 0.15	5.86 ± 3.15
	Men-AB/TUL-NP	0.06 ± 0.03	0.08 ± 0.05	0.01 ± 0.01	0.39 ± 0.18	9.36 ± 4.62
AC/TUL	AC/TUL-MP	0.12 ± 0.06	0.16 ± 0.09	0.01 ± 0.01	0.17 ± 0.08 ^#^	3.66 ± 1.59 ^#^
	AC/TUL-NP	0.05 ± 0.03	0.07 ± 0.04	0.02 ± 0.01	0.28 ± 0.19 ^#^	2.24 ± 1.58 ^#^
	Men-AC/TUL-MP	0.02 ± 0.01	0.03 ± 0.01 *	0.01 ± 0.01	0.83 ± 0.29 *	13.49 ± 5.54 *^,^
	Men-AC/TUL-NP	0.03 ± 0.01	0.02 ± 0.01 *	0.01 ± 0.01	0.75 ± 0.14 *	11.19 ± 5.15

The compositions of ointment containing TUL-MPs and TUL-NPs are presented in Table 1. *n* = 6–9. * *p* < 0.05 vs. MP formulation for each category. ^#^ *p* < 0.05 vs. MP formulation with *l*-menthol for each category.

## Data Availability

Not applicable.
